# Can internal mammary lymph nodes irradiation bring survival benefits for breast cancer patients? A systematic review and meta-analysis of 12,705 patients in 12 studies

**DOI:** 10.1186/s13014-021-01772-y

**Published:** 2021-02-23

**Authors:** Sicong Jia, Zhikun Liu, Jun Zhang, Chenguang Zhao, Longyu Zhu, Jie Kong, Huina Han, Yuguang Shang, Dongxing Shen, Xuejuan Duan

**Affiliations:** 1 Department of Radiotherapy, The Fourth Hospital of Hebei Medical University/Hebei Cancer Hospital, Shijiazhuang, 050000 China; 2 Department of Oncology, Graduate School, Hebei Medical University, Shijiazhuang, 050000 China

**Keywords:** Breast neoplasm, Internal mammary lymph node, Radiotherapy, Meta-analysis

## Abstract

**Objective:**

To evaluate the effect of prophylactic irradiation of internal mammary lymph nodes in breast cancer patients.

**Methods:**

The computer searched PubMed, EMBASE, Web of science, CNKI, Wanfang Medical Network, the Chinese Biomedical Literature Database to find clinical studies on internal mammary lymph node irradiation (IMNI) in breast cancer. The quality of the included literature was evaluated according to the Newcastle–Ottawa scale. Stata14 software was used for meta-analysis.

**Results:**

A total of 12,705 patients in 12 articles were included for meta-analyzed. Compared with patients who unirradiated internal mammary lymph nodes (non-IMNI), the risk of death for patients after IMNI was reduced by 11% (HR 0.89, 95% CI 0.79–1.00, P = 0.0470); DFS of group mixed N_+_ patients (high risk group) was significantly improved after IMNI (HR 0.58, 95% CI 0.49–0.69, P < 0.001). Further subgroup analysis shows that compared with non-IMNI, DFS was significantly increased in N_1_or ypN_1_ subgroup (HR 0.65, 95% CI 0.49–0.87, P = 0.003) and N_2_or ypN_2_ subgroup (HR 0.51, 95% CI 0.37–0.70, P < 0.001) after IMNI, but there was no statistical difference in DFS between the IMNI and non-IMNI groups in N_0_ subgroup (HR 1.02 95% CI 0.87–1.20, P = 0.794) and N_3_ or ypN_3_ subgroup (HR 0.85, 95% CI 0.49–1.45, P = 0.547). No serious incidents were reported in all the included studies, and most of the acute and late side effects were mild and tolerable.

**Conclusion:**

Under modern radiotherapy techniques, IMNI can safely and effectively bring clinical benefits to N_1–2_ breast cancer patients, but its role in N_0_, N_3_ breast cancer patients remains to be further studied.

## Introduction

Breast cancer is the most common malignant tumor among women in the world. The number of its new cases is up to 2.08 million every year, and the incidence rate is still on the rise, which is a serious threat to women's health [1]. At present, for the treatment of breast cancer patients with high risk of recurrence and metastasis, international authoritative diagnosis and treatment guidelines recommend postoperative adjuvant radiotherapy for chest wall and regional lymph nodes (RLNs) in order to obtain higher local control rate and survival rate [2–4]. Although RLNs irradiation in patients with high-risk breast cancer is strongly recommended in international guidelines, daily clinical practice often does not fully reflect these recommendations due to clinical needs that require a tailored risk-adapted radiotherapy approach [5]. Moreover, in the Radiation Oncologists' modern vision, some topics (including internal mammary irradiation) represent open fields for discussion partly linked to the lack of scientific evidence that can effectively answer the specific clinical question and partly linked to the risk of exposing the patient to unjustified side effects. In this context, as the first stop of breast lymphatic drainage [6], the internal mammary lymph nodes (IMLNs), unlike supraclavicular lymph nodes, are not routinely irradiated because of radiotherapy increases the cardiopulmonary extra dose and its uncertain clinical efficacy. There is also great controversy about the standard of internal mammary lymph nodes irradiation (IMNI) in different centers [7]. In recent years, the improvement of imaging technology making the detection rate of IMLNs metastasis has been significantly raised [8]; the rapid development of systemic treatment has greatly reduced the risk of distant metastasis [9]; technological and technical improvements in modern radiotherapy, such as IMRT or VMAT, have become widespread available in clinical practice, allowing respect 3D-CRT: a better dose distribution within the target volume (in terms of homogeneity) and a reduction in high doses to healthy tissues and organs at risk [10]. These changes may allow patients to benefit more from IMNI. There is ongoing debate about whether or not IMLNs should be irradiated. Therefore, this study included the literature on whether or not IMLNs were irradiated as an intervention measure to conduct meta-analysis, in an attempt to provide a reliable basis for the treatment of breast cancer patients.

## Material and methods

This meta-analysis was conducted according to the preferred reporting items for systematic reviews and meta-analyses (Supporting information: PRISMA Checklist).

### Literature retrieval strategy

The computer searched PubMed, EMBASE, Web of Science, CNKI, Wanfang Medical Network, the Chinese Biomedical Literature Database to find clinical studies on IMNI. Literature retrieval was carried out by the combination of subject words and free words. Take PubMed as an example: ("Breast Neoplasms"[Mesh] OR "breast neoplasm"[Title/Abstract] OR "breast tumor*"[Title/Abstract] OR "breast cancer"[Title/Abstract] OR "mammary cancer*"[Title/Abstract] OR "breast malignant neoplasm*"[Title/Abstract] OR "breast malignant tumor*"[Title/Abstract] OR "breast carcinoma*"[Title/Abstract]) AND ("internal mammary lymph node*"[Title/Abstract] OR "internal mammary lymph node areas"[Title/Abstract] OR "internal mammary lymph node chain*"[Title/Abstract] OR "internal mammary node*"[Title/Abstract] OR "internal mammary area"[Title/Abstract] OR "internal mammary lymph chain"[Title/Abstract]) AND ("Radiotherapy"[Mesh] OR "radiotherapies"[Title/Abstract] OR "radiation therap*"[Title/Abstract] OR "radiation treatment*"[Title/Abstract] OR "irradiation"[Title/Abstract]).

### Literature inclusion criteria

Study type: Clinical controlled study. Research object: (1) all included cases were pathologically confirmed breast cancer (regardless of surgical and systemic treatment modes); (2) All patients without other tumors; (3) No distant metastasis or other complications. Intervention measure: IMNI or not. Observation indicators: The main observation indicators are overall survival (OS) and disease-free survival (DFS). The secondary observation indicator is the acute and late side effects of IMNI.

### Literature exclusion criteria

(1) Summary of the meeting, literature review, letter, case report and degree paper; (2) The characteristics of patients do not meet the inclusion criteria; (3) The same study, such as different follow-up time or repeated reports; (4) Studies in which indicator information is incomplete or indicator information cannot be obtained by common methods; (5) Studies with follow-up time less than 5 years.

### Literature screening, data extraction and quality evaluation

Six researchers read the literature independently according to the inclusion and exclusion criteria, negotiated and resolved the disagreement, and extracted the relevant data in the literature. The content of data extraction includes: hazard ratios (HR) with 95% confidence limits (CI) for OS, DFS from literature. Due to the large number of retrospective cohort studies were included in this study, the Newcastle–Ottawa Scale (NOS) was used to evaluate the quality of the literature. The contents of the evaluation included: (1) Representativeness of the exposed Cohort. (2) Selection of the non-exposed cohort. (3) Ascertainment of exposure. (4) Demonstration that outcome of interest was not present at start of study. (5) Comparability of cohorts on the basis of the design or analysis. (6) Assessment of outcome. (7) Adequacy of the length of the follow up for the detection of the outcomes of interest. (8) Adequacy of follow up of cohorts. (9) Assessment of outcome. A score of 0–5 is regarded as low-quality literature, and more than 6 as high-quality literature.

### Statistical analysis

Stata (version 14) software was used for meta-analysis. First of all, this study carried on the heterogeneity test. If I^2^ < 50% and P < 0.1, it means that there is a low heterogeneity among studies. So the fixed-effect model was used for analysis; if I^2^ > 50% and P > 0.1, it means that a high heterogeneity among studies. We carried out subgroup analysis or meta regression to the factors that may lead to heterogeneity. If there was statistical heterogeneity but no clinical heterogeneity among studies, random effect model analysis was used. At the same time, this study conducted subgroup analysis on the key prognostic factors such as the status (N_0_, N +) of axillary lymph nodes (ALNs) and tumor location (medial/central, lateral), which further analyzed the specific effect of IMNI under the influence in these factors. The results are considered to be statistically different, if P < 0.05. Egger’s test was used to evaluate the potential publication bias of the study. It is considered that the publication bias is small, if P > 0.1. Finally, the sensitivity test was carried out by eliminating the literature one by one. If the literature is excluded one by one, there is no large deviation in the results, which means that the results are robust.

## Results

### Literature retrieval results

**Fig. 1:**
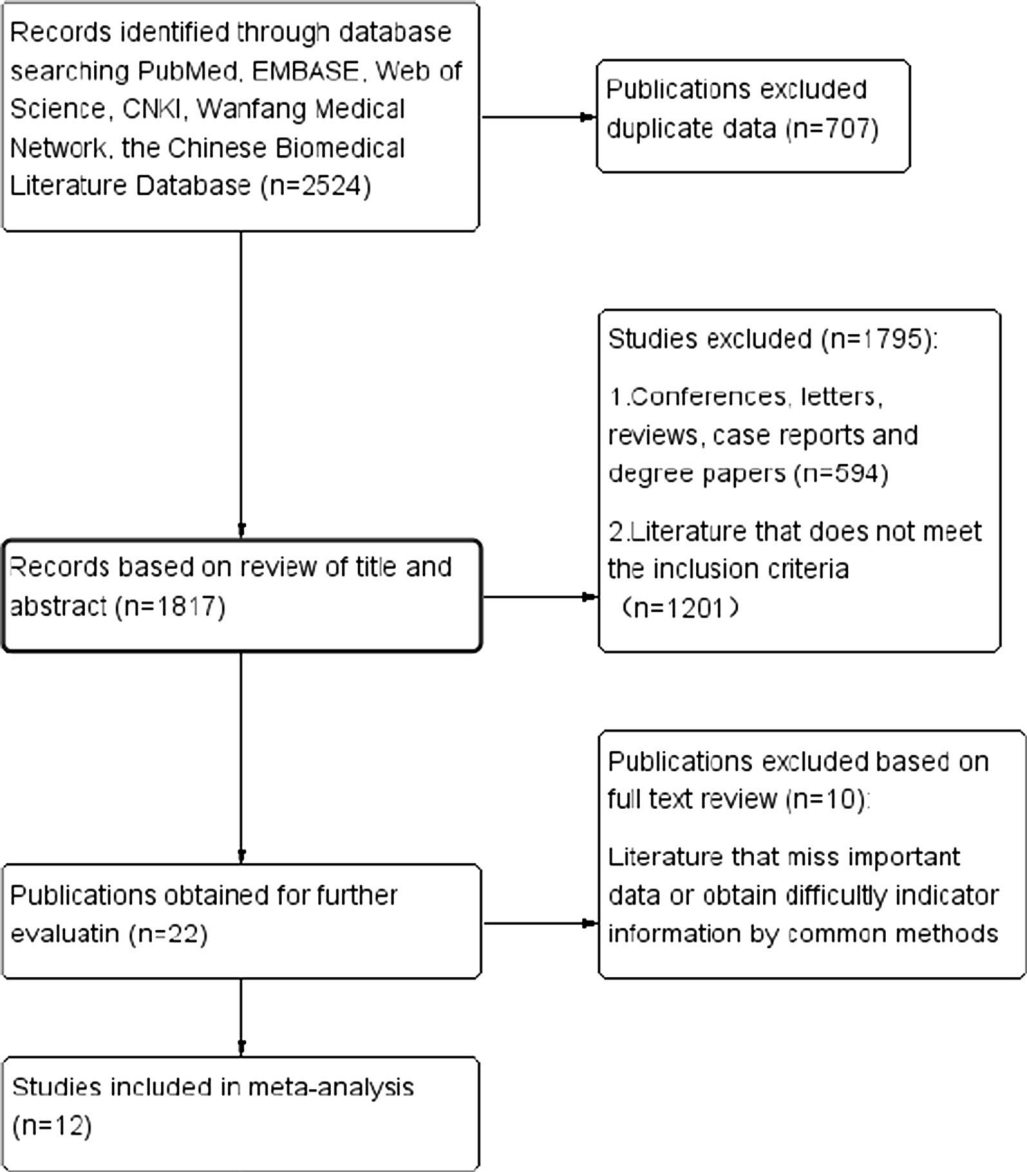
Flow diagram

A total of 1925 English literature were retrieved and 599 Chinese literature was retrieved. 707 duplicate articles were excluded. By reading the title and abstract of the literature, 594 conferences, letters, reviews, case reports and degree papers and 1201 articles that did not met the inclusion criteria were excluded. Among them, two classic randomized clinical studies, MA.20 [11] and EORTC22922-10925 [12], were excluded because they discussed RNI (supraclavicular, infraclavicular and internal mammary region) or not, which was not completely consistent with our studied on IMNI or not. A total of 22 papers passed the preliminary screening. After reading the full text, 10 papers were excluded due to missing important data or obtain difficultly indicator information by common methods. Finally, a total of 12,705 patients in 12 articles were included for meta-analyzed (Fig. 1).

### Basic characteristics and quality evaluation of included studies

The 12 included studies [13–24] were all English literature, including 9 retrospective cohort studies (RCS) [13, 14, 16–20, 22, 24], 1 prospective cohort study (PCS) [15], 1 non-randomized controlled trial (NRCT) [23], and 1 randomized controlled trial (RCT) [21]. All studies used IMNI or not as an intervention measure. Among them, 10,905 patients (86%) received chest wall / breast + supraclavicular and infraclavicular region ± IMLNs irradiation, and only 14% omitted supraclavicular and infraclavicular region irradiation because of N_0_ or treatment time. In terms of surgical treatment, patients in 4 studies [17, 18, 20, 21] underwent mastectomy, patients in 2 studies [19, 24] underwent breast-conserving surgery, and patients in 6 studies [13–16, 22, 23] underwent mastectomy or breast-conserving surgery. Most patients underwent standardized systemic treatment (Table 1).

**Table 1:** Basic characteristics of included studies

References	Country	Design	Recruitment period	Number of patients		Inclusion criteria	HR (lower CI-upper CI)		Follow-up time (month)
				IMNI	Non-IMNI		OS	DFS	
Wang [13]	China	RCS	2007–2010	390	482	pBC	0.82 (0.59–1.15)	0.54 (0.41–0.72)	98
Luo [14]	China	RCS	2005–2013	236	261	Stage II–III	0.62 (0.37–1.03)	0.65 (0.46–0.93)	64
Thorsen [15]	Denmark	PCS	2003–2007	1492	1597	N +	0.82 (0.72–0.94)	–	107
Kim [16]	Korea	RCS	2001–2009	284	237	Stage II–III	0.51 (0.26–1.01)	0.58 (0.34–1.00)	71
Yadav [17]	India	RCS	1978–1996	153	166	Stage II–III	0.89 (0.60–1.30)	0.35 (0.22–0.55)	203
Aleknavičius [18]	Lithuania	RCS	1987–1997	165	268	Tumor in central/medial	0.75 (0.55–1.01)	0.67 (0.46–0.99)	102
Courdi [19]	France	RCS	1975–2008	489	1141	N −	0.99 (0.81–1.22)	1.04 (0.88–1.23)	154
Chang [20]	Korea	RCS	1994–2002	197	199	Stage II–III	0.91 (0.64–1.28)	0.7 (0.52–0.96)	98
Hennequin [21]	France	RCT	1991–1997	672	662	N + Tumor in central/medial	0.95 (0.80–1.13)	–	103
Olson [22]	Canada	RCS	2001–2006	1000	1413	N + T3/4N0	0.95 (0.78–1.15)	–	74
Stemmer [23]	Israel	NRCT	1994–1998	67	33	Stage II–III	0.48 (0.19–1.21)	0.44 (0.18–1.08)	77
Obedian [24]	America	RCS	1970–1990	535	411	Breast conservation surgery	1.56 (1.10–2.22)	–	156

*RCS* retrospective cohort study, *PCS* prospective cohort study, *NRCT* non-randomized controlled trial, *RCT* randomized controlled trial

In the included literature, 11 articles [13–18, 20–24] with ≥ 6 points were rated as high-quality articles, and 1 article [19] with a quality score of 5 points was low quality. Among them, 2 articles [15, 19] had high risk of cohort representation, 1 article [18] had insufficient follow-up time, and 10 articles [13, 14, 16–22, 24] did not describe the loss of follow-up. (Table. 2).

**Table 2:** quality evaluation of included studies

References	Representativeness of the exposed cohort	Selection of the non-Exposed cohort	Ascertainment of exposure	Demonstration that outcome of interest was not present at start of study	Comparability of cohorts on the basis of the design or analysis	Assessment of outcome	Was follow-up long enough for outcomes to occur	Adequacy of follow up of cohorts	Score
Wang [13]	*	*	*	*	**	*	*	–	8
Luo [14]	*	*	*	*	**	*	*	–	8
Thorsen [15]	–	–	*	*	*	*	*	*	6
Kim [16]	*	*	*	*	**	*	*	–	8
Yadav [17]	*	*	*	*	*	*	*	–	7
Aleknavičius [18]	*	*	*	*	*	*	–	–	6
Courdi [19]	–	–	*	*	*	*	*	–	5
Chang [20]	*	*	*	*	*	*	*	–	7
Hennequin [21]	*	*	*	*	*	*	*	–	7
Olson [22]	*	*	*	*	*	*	*	–	7
Stemmer [23]	*	*	*	*	*	*	*	*	8
Obedian [24]	*	*	*	*	*	*	*	–	7

“*” means yes; “–” means no

### Results of meta-analysis

**Fig. 2:**
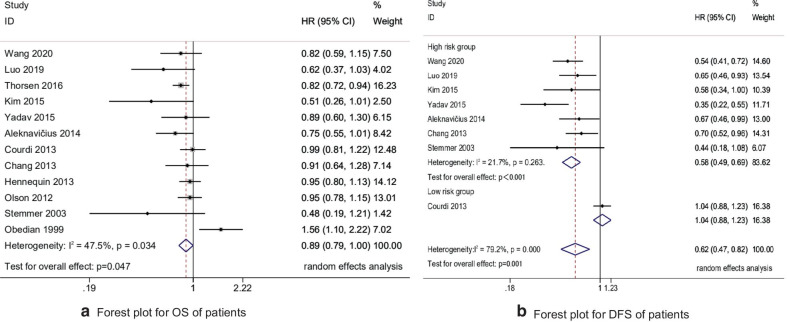
Forest plot for OS and DFS of patients

The HR data of OS could be extracted from 12,705 patients in 12 studies [13–24]. First of all, the heterogeneity test was performed, I^2^ = 47.5% and P = 0.034. The heterogeneity of the study was at a critical value. We conducted a meta-regression analysis on factors such as the follow-up time, the date of patients were treated, and the study type, and the results showed that none of the above three factors caused significant heterogeneity (P_follow-up_ = 0.243, P_date_ = 0.940, P_type_ = 0.656). Therefore, a random effects model is used for meta-analysis. Compared with patients who unirradiated internal mammary lymph nodes (non-IMNI), the risk of death for patients after IMNI was reduced by 11% (HR 0.89, 95% CI 0.79–1.00, P = 0.047) (Fig. 2a). The HR data of DFS could be extracted from the patients in 8 studies [13, 14, 16–20, 23]. First of all, the heterogeneity test was carried out, P < 0.001 and I^2^ = 79.2%. The heterogeneity of the study was large. We performed meta-regression analysis on the patient's ALNs status (N_0_, Mixed N_+_) based on clinical characteristics. The results show that the N_0_ patient was a source of heterogeneity (P = 0.011), so we conducted the subgroup analysis and the random effects model. Compared with non-IMNI patients, DFS of group mixed N_+_ patients (high risk group) was significantly improved after IMNI (HR 0.58, 95% CI 0.49–0.69, P < 0.001) (Fig. 2b).

In the practice of diagnosis and treatment, patients in IMNI group usually have more positive lymph nodes, later staging and more high risk than patients in non-IMNI group. In order to balance the general condition of patients in the two groups, 3 studies [13, 14, 16] conducted a propensity score matching (PSM). After PSM, meta analysis showed that IMNI significantly increased OS (HR 0.63, 95% CI 0.47–0.85, P = 0.002) and DFS (HR 0.56, 95% CI 0.45–0.70, P < 0.001).

**Fig. 3:**
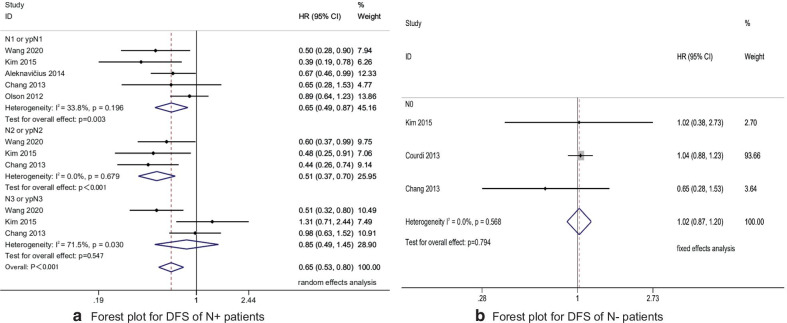
Forest plot for DFS of N + and N − patients

At the same time, we analyzed the factors such as ALNs status and tumor location. Patients with positive ALNs were divided into N_1_ or ypN_1_ subgroup, N_2_ or ypN_2_ subgroup, N_3_ or ypN_3_ subgroup. The results showed that compared with non-IMNI, DFS was significantly increased in N_1_or ypN_1_ subgroup (HR 0.65, 95% CI 0.49–0.87, P = 0.003) and N_2_or ypN_2_ subgroup (HR 0.51, 95% CI 0.37–0.70, P < 0.001) after IMNI, but the increase of DFS in N_3_ or ypN_3_ subgroup (HR 0.85, 95% CI 0.49–1.45, P = 0.547) was not statistically significant. (Fig. 3a) In addition, in the N_0_ subgroup, there was no statistical difference in DFS between the IMNI and non-IMNI groups (HR 1.02 95% CI 0.87–1.20, P = 0.794) (Fig. 3b) The location of the tumor was divided into central/medial subgroup, lateral subgroup. The results showed that after IMNI, the DFS of patients in both subgroups were significantly increased (HR_central/medial_ 0.55, 95% CI 0.40–0.78, P = 0.001; HR_lateral_ (0.77, 95% CI 0.61–0.97, P = 0.025).

### Acute and late side effects

As the types of acute and late side effects reported in various studies were not the same, and the relevant data could not be extracted completely, the acute and late side effects were analyzed by qualitative analysis instead of quantitative analysis. Two studies [13, 16] reported that the incidence of radiation pneumonitis/pulmonary fibrosis in the IMNI group was higher than that of the non-IMNI group. Different from these two studies, Yadav et al. [17] reported that there was no significant diversity in the incidence of radiation pneumonia between IMNI and non-IMNI groups. And the severity of the reaction was relatively mild, grade mostly was 1 to 2. Although Luo et al. [14] reported that the mean heart dose (MHD) of patients treated with IMNI was higher than that of patients treated with non-IMNI, especially in patients with left breast cancer (IMNI_left_ VS non-IMNI_left_ 9.1 Gy VS 4.4 Gy, P < 0.001), five studies [15–18, 21] of all included studies comparing the cardiac toxicity between IMNI and non-IMNI groups showed that there was no significant difference in cardiovascular injury. 1 study [23] reported that the skin reaction of patients in IMNI group was slightly more than that in non-IMNI group. In addition, Courdi et al. [19] reported that the IMNI group seemed to have a higher incidence of contralateral breast cancer in the long term (HR 2.47, 95% CI 1.23–4.95).

### Publication bias and sensitivity test

**Fig. 4:**
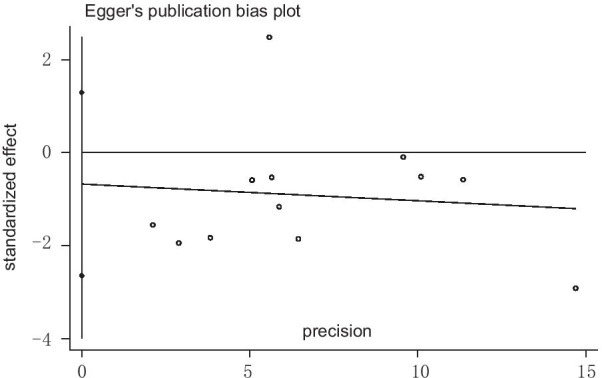
Eggers publication bias plot

Egger's test showed that P = 0.462, suggesting that publication bias was small. (Fig. 4) By eliminating the literature one by one, we found that the research results had not changed fundamentally, which showed that the results of meta-analysis were relatively stable.

## Discussion

Breast cancer is the most common malignant tumor in women [1]. At present, postoperative radiotherapy is the standard treatment for patients with high-risk breast cancer [2–4]. But the IMNI has always been controversial because of extra cardiopulmonary dose, especially left breast cancer, and its uncertain clinical efficacy. In 2014, EBCTCG [25] conducted a meta-analysis of 8135 patients who underwent chest wall and RLNs (including IMLNs) irradiation. Radiotherapy reduced the local recurrence rate and mortality in ALNs-positive patients, which established the importance of regional lymph node irradiation (RNI) for local control and long-term survival. During the 10-year follow-up of the MA.20 and EORTC22922/10,925 clinical trials [11, 12], the RNI group respectively increased disease-free survival by 5% and 3%, compared with the control group (non-RNI group); decreased the distant metastasis rate by 3.9% and 3%, respectively. However, there was no significant difference in survival rate, which was only slightly higher than that in the control group. Budach et al. [26] conducted a meta-analysis that included the above two studies and the French Lyon study, and the results showed that additional regional radiotherapy to the internal mammary and medial supraclavicular lymph nodes statistically significantly improved DFS, DMFS, and OS in stage I–III breast cancer. But, it is worth noting that these trials irradiated both supraclavicular and internal mammary areas, which were impossible to determine how much of the clinical benefits could be attributed to IMNI. Recently, with the deepening of research on IMLN, there has been a new understanding of IMNI problem. Therefore, this study included the literature of IMNI or not as an intervention for meta-analysis. The results showed that IMNI could increase OS and DFS of breast cancer patients, and through the forest plot for OS of patients, it can be seen that with the passage of time, the HR gradually shifts to the direction of IMNI. This may be related to the fact that the rapid development of radiotherapy technology from two-dimensional to three-dimensional era has significantly reduced the cardiopulmonary dose and improved accuracy of target volume since the beginning of the twenty-first century.

Lv et al. [27] reported a meta-analysis on IMNI in patients with stage I to III breast cancer, including 7 studies (6835 patients). In contrast to our study, the meta-analysis showed that there was no significant difference in 5-year survival rate (OR 1.08, 95% CI 0.93–1.27), 10-year survival rate (OR 0.86, 95% CI 0.55–1.35) and 10-year disease-free survival rate (OR 1.20, 95% CI 0.99–1.45) between IMNI and non-IMNI groups. The study included numerous early literature, the survival of patients might be affected by the backward radiotherapy technology in the early stage. In addition, the study did not conducted a subgroup analysis, which didn’t showed that whether IMNI had different effects on different subgroups. Wang et al. [13] reported that IMNI could significantly improve DFS in patients with clinical stage I–II (HR 0.277, 95% CI 0.134–0.573, P = 0.001; HR 0.507, 95% CI 0.352–0.726, P < 0.001), but there was no significant difference among patients in stage III. Kim et al. [16] made it clear that except for triple negative breast cancer (HR 0.5, 95% CI 0.250–1.000), patients with other molecular typing did not increase DFS benefit from IMNI. We further analyzed the specific effect of IMNI under the influence on ALNs status and tumor location. Analysis showed that IMNI especially increased the DFS of patients in N_1–2_ groups, but there was no statistical difference in DFS improvement among patients in N_0_, N_3_ groups. N_0_ patients had a lower risk of recurrence and metastasis and a better prognosis. On the contrary, due to their long-term survival, the late side effects of IMNI will be highlighted. Compared with N_1–2_, N_3_ patients had a later stage and easier recurrence and metastasis, the clinical benefit brought by IMNI might be diluted by N_3_′ s highly malignant biological behavior.

It is worth noting that in the N3 subgroup, unlike the results of two other earlier studies, recently published studies by Wang et al. showed that IMNI significantly increased DFS in patients. As we all know, the systemic treatment of breast cancer patients has developed rapidly since 2010. HERA study had shown that one-year adjuvant therapy with trastuzumab can reduce the recurrence rate in breast cancer patients with overexpression of HER2 protein or gene amplification (determined to be HER2 positive) [28]. APHINITY study reported that in the cohort of patients with node-positive disease, the 3-year rate of invasive-disease-free survival was 92.0% in the pertuzumab group, as compared with 90.2% in the placebo group (HR 0.77, 95% CI 0.62- 0.96, P = 0.02) [29]. For the endocrine therapy of breast cancer patients, the results of TEXT and SOFT studies clearly suggested that OFS + AI treatment for young and high-risk early breast cancer patients can get clinical benefits [30]. In addition, traditional standard chemotherapy drugs have also explored new treatment methods. The efficacy and safety of metronomic chemotherapy, such as capecitabine, vinorelbine and cyclophosphamide, had been verified. The treatment mode also changed from single adjuvant chemotherapy to neoadjuvant chemotherapy and then to neoadjuvant chemotherapy combined with targeted therapy and endocrine therapy based on different molecular subtypes. Therefore, we can see that the current strong systemic treatment of breast cancer has significantly reduced the recurrence and metastasis rate of patients, which provides a strong guarantee for the efficacy of local treatment of breast cancer. Therefore, we can not rule out the possibility that the clinical efficacy of IMNI will be concealed by non-standard and incomplete systemic treatment in patients with overloaded tumors in the past. In the future, we still need to further explore whether IMNI can bring survival benefits to patients with overloaded tumors under modern adequate systemic therapy.

Our work mainly analyzed the impact of IMNI on the survival of breast cancer patients, and most of the patients received chest wall / breast + supraclavicular and infraclavicular region ± IMLNs irradiation. In the radiation oncologists' modern vision, survival of low-risk patients such as N_0_ would not been significantly affected by RNI after being strictly judged by the physician. Therefore, we inevitably mixed a small number of patients who were exempted from supraclavicular and infraclavicular region irradiation because of low risk such as N_0_. Therefore according to authority guidelines, we might believe that these patients would not have a significant impact on the results of our overall observation.

In terms of acute and late side effects, although Courdi et al. [19] reported that the IMNI group seemed to have a higher incidence of contralateral breast cancer in the long term (HR 2.47, 95% CI 1.23–4.95), it couldn’t be excluded that these results suffered the bias of comparing populations included over a long period of time, therefore with unequal follow-ups. What’s more, Patients were enrolled from 1975 to 2008, Some patients might suffer suboptimal past radiotherapy where imprecise field shapes and unsophisticated dosimetry might have delivered unnecessary radiation to the opposite breast. In addition, the risk of recurrence in N_0_ patients is low, and the role of IMNI in this low-risk population is not obvious, and it can not be ruled out that these patients are overtreated. Therefore, under modern radiotherapy technology, the impact of IMNI on the occurrence of contralateral breast cancer needs more evidence to prove. At present, the wide application of deep inspiration breath hold technology has significantly reduced the dose of heart [31]. For complicated plans or patients with poor chest wall anatomy, new technologies such as VMAT and TOMO can also significantly reduce the cardiopulmonary dose of patients [32, 33]. In addition, proton radiotherapy has shown a good ability to reduce the cardiopulmonary dose [34, 35], and conditional centers can carry out proton radiotherapy to enhance the protection of organs at risk. It can be seen that although IMNI increases the dose of heart, lung and other important organs, we can still control it at a low level by modern technical means. All in all, no serious incidents were reported in all the included studies. Most of the acute and late side effects were mild and tolerable.

In clinical studies, only the French Lyon study [21] was a randomized III phase study of IMNI, so most of the literature included was RCS. Although meta regression analysis of study type, follow-up time as variables showed that its did not affect the results of the study, the RCS must have certain quality limitations. In addition, the statistical effectiveness of evidence-based medicine was inevitably reduced due to the small number of studies included in subgroups with different lymph node status. On the other hand, tumor biological characteristics, patient staging, radiotherapy techniques and radiation dose and other factors might also have an impact on OS, DFS of patients, but only a small number of studies have reported these subgroups and Even if there were articles describing these factors, it was difficult to obtain specific data such as the number of people with different factors, which made it impossible for us to make a quantitative analysis. The exploration of the influence of these factors on the efficacy of IMNI is also the direction of development in the future. Therefore, we still look forward to the results of multicenter RCT on IMNI to further guide the treatment of breast cancer patients. At present, “Postmastecomy Internal Mammary Nodal Irradiation for High-risk Breast Cancer Patients” [36] led by the Cancer Hospital Chinese Academy of Medical Sciences is enrolling patients, and the corresponding trial results are expected to be published in 2024. We believe that with a series of randomized controlled trials published, the debate on the treatment of IMLNs in breast cancer patients will be gradually ceased.

## Conclusion

Under modern radiotherapy techniques, IMNI can safely and effectively bring clinical benefits to N_1–2_ breast cancer patients, but its role in N_0_, N_3_ breast cancer patients remains to be further studied.

## Data Availability

All data generated or analyzed during this study are included in this manuscript and its Additional files.

## Abbreviations

IMNI: Internal mammary lymph node irradiation
RLNs: Regional lymph nodes
IMLNs: Internal mammary lymph nodes
HR: Hazard ratios
OS: Overall survival
DFS: Disease-free survival
ALNs: Axillary lymph nodes
RCS: Retrospective cohort study
PCS: Prospective cohort study
NRCT: Non-randomized controlled trial
RCT: Randomized controlled trial
PSM: Propensity score matching
MHD: Mean heart dose
RNI: Regional lymph node irradiation
